# Maternal Preeclampsia and Androgens in the Offspring around Puberty: A Follow-Up Study

**DOI:** 10.1371/journal.pone.0167714

**Published:** 2016-12-19

**Authors:** Ingvild V. Alsnes, Imre Janszky, Bjørn O. Åsvold, Inger Økland, Michele R. Forman, Lars J. Vatten

**Affiliations:** 1 Department of Obstetrics and Gynecology, Stavanger University Hospital, Stavanger, Norway; 2 Department of Public Health, Faculty of Medicine, NTNU, Norwegian University of Science and Technology, Trondheim, Norway; 3 Department of Endocrinology, St. Olavs Hospital, Trondheim University Hospital, Trondheim, Norway; 4 Department of Nutritional Sciences, University of Texas at Austin, Austin, Texas, United States of America; John Hopkins University School of Medicine, UNITED STATES

## Abstract

**Background:**

Children born after preeclampsia may have a dominant androgen profile in puberty compared with other children. Circulating androgen concentrations at 11–12 years of age were compared between offspring born after preeclampsia, and children whose mothers did not have preeclampsia.

**Methods:**

A total of 611 mother-offspring pairs were followed up 11 (daughters) or 12 (sons) years after birth: 218 pairs in the preeclampsia group, and 383 pairs without preeclampsia. Circulating total testosterone, androstenedione, dehydroepiandrosterone sulfate (DHEAS), and insulin-like growth factor I (IGF-I) were measured in the children. In boys, testicular volume was also measured.

**Results:**

Among girls born after preeclampsia, DHEAS concentrations were higher than in unexposed girls (p<0.001), however, girls born after preeclampsia with severe features had the lowest DHEAS levels. In contrast, testosterone concentrations were highest in girls born after preeclampsia with severe features, both compared to other girls in the preeclampsia group, and compared to unexposed girls (p<0.001). For boys, testosterone concentrations were higher in the preeclampsia group compared with unexposed boys (p<0.001), and boys born after preeclampsia with severe features had the lowest concentrations of DHEAS. Compared with unexposed boys, testicular volume (p = 0.015) and IGF-I (p = 0.004) were higher for boys in the preeclampsia group, except for boys in the clinically severe preeclampsia group.

**Conclusions:**

*In utero* exposure to preeclampsia is associated with androgen hormonal patterns in early puberty that depend on clinical severity of preeclampsia and sex of the offspring. The hormonal differences may reflect different timing of pubertal development, and may have consequences for future health of the offspring.

## Introduction

Preeclampsia is a pregnancy-associated syndrome characterized by persistent hypertension and proteinuria after 20 weeks of gestation that affects 2–6% of pregnancies in the Western world.[1,2] There is convincing evidence that women with a history of preeclampsia are at increased risk of cardiovascular disease later in life, and possibly at reduced risk of breast cancer.[3–5] Whether the offspring of preeclamptic pregnancies carry similar risks has received less attention, but some studies have suggested possible associations of maternal preeclampsia with risk of cardiovascular disease and breast cancer later in life.[6,7]

It has been suggested that androgen levels are higher in preeclampsia pregnancies compared with normotensive pregnancies [8,9], and that hormonal characteristics associated with preeclampsia may be of importance for the offspring. Thus, the timing of and transition through puberty may be a period that allows the particular preeclampsia-related phenotype to appear.[10]

Large amounts of dehydroepiandrosterone-sulfate (DHEAS) and androstenedione are produced in the adrenal cortex in the fetal period, but the production of adrenal androgens decreases rapidly after birth. The production of the adrenal hormones reactivates as the adrenal cortex matures, as early as three years of age but usually from 4–6 years of age, a process that is often called the adrenarche. However, its physiological role is still unclear. DHEAS is converted to testosterone, and the gradual increase in adrenal androgens precedes the development of pubic and axillary hair growth (pubarche).[11]

In a previous analysis of data from the present study population, daughters whose mothers had preeclampsia in pregnancy tended to enter puberty through the pubarche pathway, whereas the thelarche (breast development) pathway tended to initiate puberty in daughters whose mothers did not have preeclampsia.[12] This finding could indicate that hormonal factors associated with preeclampsia influence the beginning of puberty, and partly determine the initial pubertal pathway.

Therefore, we examined hormonal status at the age of 11 (girls) and 12 years (boys) in a follow-up study of children whose mothers participated in a study at the children´s birth, where children born after maternal preeclampsia were compared with children whose mother did not have preeclampsia. The main aim of the present study was to examine whether androgen concentrations in early puberty differed between these children.

## Materials and Methods

### Study population

The present study is a follow-up of a nested case-control study among all births at Stavanger University Hospital in Norway over a three-year period (1993–95). The purpose of the original study was to assess maternal factors related to risk of preeclampsia, and to evaluate the association between different clinical manifestations of preeclampsia and fetal growth restriction. Among 12,804 consecutive singleton deliveries, 307 mothers diagnosed with preeclampsia and their offspring were compared with 619 mother-offspring pairs without preeclampsia, who were frequency-matched to the preeclampsia group by maternal age or by date of delivery.[13,14] The mother-offspring pairs were followed up when the offspring were expected to enter puberty, i.e. after approximately 11 years for mother-daughter pairs and 12 years for mother-son pairs.[15] At follow-up, 601 mother-offspring pairs attended (65% of the original study), of whom 218 mothers had a history of preeclampsia and 383 mothers did not. There were no differences in baseline characteristics, including maternal age, weight or height, and no differences in frequency or clinical severity of preeclampsia between those who attended and those who did not attend the follow-up.[16,17]

When the original perinatal study was initiated, the diagnostic criteria for preeclampsia were defined according to those used in the Collaborative Low-dose Aspirin Study in Pregnancy (CLASP).[18] In addition, preeclampsia cases were categorized according to clinical severity, as mild, moderate or severe. Thus, clinically mild preeclampsia (n = 73) was defined as a diastolic blood pressure increase of at least 25 mmHg and proteinuria 1+ on semiquantitive dipstick, whereas clinically moderate preeclampsia (n = 91) was defined as a diastolic blood pressure increase of at least 25 mmHg and proteinuria 2+ on semiquantitive dipstick. Severe preeclampsia (n = 54) was defined as a diastolic blood pressure of at least 110 mmHg, or an increase in diastolic blood pressure of at least 25 mmHg, and proteinuria 3+ on semiquantitive dipstick, or at least 500 mg/24 hours.[13] The definition of preeclampsia has evolved over time, and in a sensitivity analysis, we therefore combined clinically mild and moderate preeclampsia as one category, and kept preeclampsia with severe features as a separate category.

### Data collection

At a mean age of 10.8 (SD 1.3 months) and 11.8 years (SD 1.4 months) after delivery, mother-daughter and mother-son pairs, respectively, participated in the follow-up study that included questionnaires, a clinical examination and collection of fasting blood samples. Three trained nurses at the pediatric out-patient clinic at Stavanger University Hospital conducted the clinical examinations. Body weight was measured to the nearest 0.1 kg using an electronic scale (Seca 770, Hamburg, Germany), and height was measured to the nearest 0.1 cm, using the Harpenden stadiometer (Holtain, Crosswell, Crymych, Wales, UK), and recorded as the mean of two successive measurements. Body mass index (BMI) was calculated as weight (kg) divided by height (meter) squared, and the waist to hip ratio was calculated as the ratio of the mean value of two measurements of waist and hip circumference, recorded to the nearest 0.1 cm. Pubertal development was recorded by Tanner staging.[19,20] Tanner stage of breast, pubic hair and testicular volume were clinically assessed by trained nurses who were blinded to the mother´s preeclampsia status, and testicular volume was measured to the nearest millimeter using Prader’s orchidometer, which has shown high validity using ultrasound measurements as the reference standard.[21] Concentrations of total testosterone, androstenedione, and dehydroepiandrosterone sulfate (DHEAS) were measured by HPLC/mass spectrometry (total testosterone) or HPLC/tandem mass spectrometry (androstenedione and DHEAS). Insulin-like growth factor I (IGF-I) was measured by blocking radioimmunoassay (RIA) after acid alcohol extraction. In the mothers, androstenedione and IGF-I were also measured, and sex hormone binding globulin (SHBG) was measured by electrochemiluminescence immunoassay (ECLIA). All blood samples were analyzed at the Endocrine Sciences Laboratory of Esoterix Inc, Austin, Texas, USA and verified by repeat analysis in a subsample. The study was approved by the Regional Committee for Medical and Health Research Ethics (Norway), and by the Institutional Review Boards of the National Cancer Institute and of the University of Texas at Austin. The participants of this study provided written informed consent to participate in the study. For minors, the legal guardian of the offspring signed on their behalf. The ethics committee approved of the consent procedure.

### Statistical analyses

Using general linear models, we compared mean values of total testosterone, androstenedione, DHEAS, and IGF-I in offspring in the three maternal preeclampsia groups and in the group without preeclampsia. Variables with a skewed distribution were logarithmically transformed in the statistical analysis, but in the results, we present the mean values and SD without logarithmic transformation to allow comparison with the results of other studies. We adjusted for maternal age (5-year categories) and education (3 categories). In a supplementary analysis, we also adjusted for Tanner score and testicular volume (boys). In a sensitivity analysis, we combined clinically mild and moderate preeclampsia as one category, kept preeclampsia with severe features as a separate category, and made comparisons with children whose mothers did not have preeclampsia. All analyses were computed using SPSS version 20 and SAS version 9.3.

## Results

Characteristics of the mothers are presented in Table 1. At delivery, the mothers in the preeclampsia group were approximately 27 years of age, and mothers in the normotensive group were slightly older. Maternal body weight and BMI were higher in the clinically mild and moderate preeclampsia groups, both compared with mothers in the normotensive group and mothers who had preeclampsia with severe features. Pregnancy length was shorter in the preeclampsia group, and particularly short in the clinically severe group of preeclampsia (Table 1).

**Table 1:** Maternal characteristics according to preeclampsia status at delivery.

Preeclampsia status	No	Clinically mild	Clinically moderate	Severe features
N	383	73	91	54
	Mean±SD^d^	Mean±SD	Mean±SD	Mean±SD
Age^a^ (years)	28.5±4.8	27.7±4.5	27.3±4.5	26.8±4.8
Weight^b^ (kg)	69.7±13.1	76.8±16.4	74.3±17.8	70.2±11.6
BMI^b^^,^^c^ (kg/m^2^)	24.7±4.3	27.5±5.5	26.5±6.01	25.3±4.4
Pregnancy length (days) N	280.6±10.5	273.1±16.2	268.3±17.4	247.7±28.2
Parity^b^	3.0±0.9	3.1±0.8	2.8±0.9	2.6±0.9
	N (%)	N (%)	N (%)	N (%)
Education				
<=9 years	86 (22.8)	17 (23.3)	15 (16.9)	11 (21.2)
10–12 years	192 (50.8)	35 (47.9)	46 (51.7)	31 (59.6)
>12 years	100 (26.5)	21 (28.8)	28 (31.5)	10 (19.5)

^a^At delivery

^b^At follow-up

^c^BMI = Body mass index

^d^SD = Standard deviation

In Table 2, we compared hormone values among children who were not exposed to preeclampsia *in utero* and children whose mothers had preeclampsia. Among girls (Table 2), the highest testosterone concentrations were observed in the clinically severe preeclampsia group, followed by unexposed girls (39% lower testosterone), whereas girls in the clinically mild and moderate preeclampsia group had testosterone levels that were 82–87% lower compared with girls in the unexposed group (p<0.001). On the other hand, girls in the clinically mild and moderate preeclampsia group had the highest DHEAS concentrations, with 122–143% higher concentrations compared with girls in the unexposed group (p<0.001). In contrast, girls in the clinically severe preeclampsia group had the lowest concentrations of DHEAS (p = 0.011). We found no clear differences in androstenedione between the groups, and no clear differences in IGF-I.

**Table 2:** Hormonal differences^a^ from the reference group (no preeclampsia) among offspring at 11–12 years by exposure to preeclampsia *in utero*^b^.

Preeclampsia status	Clinically mild	Clinically moderate	Severe features
	Difference (p-value)	Difference (p-value)	Difference (p-value)
Girls (unit)			
Testosterone Total (ng/dL)	-34.3 (<0.001)	-38.2 (<0.001)	31.6 (<0.001)
DHEAS ^c^ (ug/dL)	34.0 (<0.001)	28.8 (<0.001)	-14.9 (0.011)
Androstenedione (ng/dL)	3.94 (0.949)	-2.62 (0.765)	-5.10 (0.658)
IGF-I ^d^ (ng/mL)	-1.77 (0.940)	2.22 (0.901)	-13.8 (0.605)
Boys (unit)			
Testosterone Total (ng/dL)	39.6 (0.005)	26.0 (0.04)	21.3 (<0.001)
DHEAS (ug/dL)	8.1 (0.129)	11.0 (0.072)	-53.0 (<0.001)
Androstenedione (ng/dL)	1.06 (0.366)	-0.69 (0.835)	-2.36 (0.677)
IGF-I (ng/mL)	34.7 (0.033)	37.7 (0.043)	-34.4 (0.063)
Testicular volume (mL)	1.061 (0.038)	1.572 (0.012)	-0.105 (0.861)

^a^ Adjusted for maternal age and education (least square means)

^b^See S2 Table for actual concentrations

^c^ DHEAS = Dehydroepiandrosterone sulfate

^d^ IGF-I = insulin-like growth factor 1

Among boys, testosterone concentrations were higher in the preeclampsia group, compared with unexposed boys (p<0.001). Within the preeclampsia group, however, testosterone was highest in boys whose mother had clinically mild (63% higher) or moderate (42% higher) preeclampsia, compared to unexposed boys. Boys who were born after clinically severe preeclampsia had the lowest concentrations of DHEAS (Table 2), with 90% lower concentrations than other boys in the preeclampsia group, and their DHEAS was also lower than in unexposed boys (p<0.001). Similar to the findings among girls, there were no clear differences in androstenedione concentrations, however, concentrations of IGF-I were 13–15% higher among boys in the clinically mild and moderate preeclampsia groups, compared to unexposed boys (p = 0.004). In an analysis of testicular volume, boys in the mild and moderate preeclampsia groups had 21–31% higher testicular volume than boys whose mothers did not have preeclampsia (p = 0.015). On the other hand, boys in the clinically severe preeclampsia group had slightly lower testicular volume compared to unexposed boys (Table 2, S3 Table). In a sensitivity analysis, combining clinically mild and moderate preeclampsia into one category, the differences described above were not substantially altered, but the precision of the differences was slightly strengthened due to the higher number of participants within the combined preeclampsia category (S4 Table).

Since the hormonal differences could be attributed to differences in pubertal timing, and maybe correlated with differences in Tanner stage, height or BMI (S1 Table), we adjusted for these factors in the statistical analysis. However, the differences in total testosterone and DHEAS concentrations remained essentially unchanged after these adjustments (results not shown). Also, adjustment for testicular volume among the boys did not substantially influence the striking hormonal differences.

## Discussion

In this follow-up study of children at 11 (girls) and 12 (boys) years of age, androgen levels differed markedly according to maternal preeclampsia status at the children´s birth. Testosterone concentrations in girls showed a seemingly paradoxical pattern: in the clinically mild or moderate preeclampsia groups testosterone concentrations were much lower than in unexposed girls, whereas girls in the clinically severe preeclampsia group had substantially higher testosterone. In contrast, all boys in the preeclampsia group had higher testosterone concentrations compared to unexposed boys. We also found intriguing patterns for DHEAS concentrations: whereas girls in the clinically mild or moderate preeclampsia groups had much higher concentrations than unexposed girls, girls born after clinically severe preeclampsia had substantially lower DHEAS. For boys in the clinically severe preeclampsia group, DHEAS was also low, whereas in the mild and moderate preeclampsia groups, DHEAS did not substantially differ from that of unexposed boys.

Tenhola et al. found no association between exposure to preeclampsia *in utero* and DHEAS concentrations at 12 years of age, whereas low birth weight, which may occur in clinically severe preeclampsia, was associated with relatively high DHEAS concentrations at 12 years of age.[22–24]

It is a strength of our study that the participants were recruited among all births that occurred over three years in the general population, and that the follow-up 11–12 years after birth included 65% of the original participants, who were recruited at birth. The standardized clinical information collected at follow-up, including fasting blood samples collected by phlebotomists, and the high-standard laboratory specializing in endocrine measurements, are also strong features of this study. The narrow range in age at the follow-up examination reduced the variability within our data, as hormonal levels may strongly vary during puberty.

Preeclampsia was categorized according to strict diagnostic criteria, similar to the criteria used in a clinical trial (CLASP) that was conducted around the time of birth of the participants of our study. Preeclampsia was further classified as clinically mild, moderate or severe based on available information from the hospital records.[18,25] The classification of preeclampsia has evolved over the last 20 years, and in a sensitivity analyses, we combined clinically mild and moderate preeclampsia as one category, and kept preeclampsia with clinically severe features as a separate category. However, the results did not substantially differ from those of the main analyses.

The nurses who were responsible for the data collection at follow-up, were blinded to maternal preeclampsia status at delivery. Thus, the abstraction of medical records as a basis for the diagnosis of preeclampsia, and the rigorous collection of clinical data make it unlikely that our findings can be attributed to biased information. Nonetheless, in the invitation letter, the intention to assess body size and sexual maturation was mentioned, and we cannot exclude the possibility that this information could have influenced the mothers’ and children’s choice to attend the study. For example, it is conceivable that eligible participants who declined to participate may differ from those who attended with respect to anthropometric and developmental factors.[16] In this regard, however, it is reassuring that attendance at follow-up did not substantially differ between the exposed and unexposed groups, and that the proportion of attendees among the exposed and unexposed groups did not differ by perinatal characteristics.

It is a limitation of the study that all hormones relevant for pubertal status were not measured, and that repeated hormonal measurements throughout puberty have not been conducted. Therefore, we could not address whether the observed differences by preeclampsia status persisted during and beyond puberty. We have not found other longitudinal or more detailed studies on preeclampsia and sex hormones that could help us explain why the associations differed across hormones, preeclampsia group, and sex. As we have not found comparable studies to help the interpretation, we are hesitant to speculate on the matter.

In the analysis, we adjusted for Tanner stage (girls and boys) and testicular volume (boys) to evaluate whether differences in the timing of puberty could explain our findings, but the results were not substantially altered. However, Tanner staging may not capture subtle, but important differences in pubertal development, especially when the testes are small and the Tanner stage is low. Moreover, we cannot exclude the possibility of residual confounding related to developmental factors that we did not measure.

Thelarche is usually the initial sign of puberty in girls, as shown in a 5-year longitudinal population study in Denmark.[26] However, in a previous analysis of data from the present study population, pubarche tended to precede thelarche in daughters born after a preeclamptic pregnancy, whereas in daughters of a normotensive pregnancy, thelarche appeared first, as usually expected, suggesting a stronger androgenic influence at the beginning of puberty in the preeclampsia group.[12] It has been suggested that androgen production in the adrenals may start relatively early for girls born after pregnancies complicated by mild or moderate preeclampsia, and that adrenal activation may be attributed to a relative excess of fetal glucocorticoids.[27] Possibly, the higher levels of DHEAS among girls born after clinically mild or moderate preeclampsia in our study may indicate that these girls entered adrenarche relatively early, whereas the clinically severe preeclampsia group, characterized by low DHEAS, the adrenarche may occur relatively late. These differences by clinical severity of preeclampsia may also point to different disease risk in adulthood. Thus, an early age at adrenarche has been linked to functional ovarian hyperandrogenism, suggesting increased risk of polycystic ovarian syndrome (PCOS) and insulin resistance later in life.[28–30]

We found that boys in the clinically mild and moderate preeclampsia groups had markedly higher concentrations of total testosterone, higher testicular volume and higher concentrations of IGF-I than other boys. Since IGF-I stimulates steroidogenesis, the interplay between IGF-I and hyperinsulinism may indicate that early onset androgen excess could be associated with metabolic disturbances later in life.[30] Of relevance to this, Ruder et al. have suggested that intrauterine androgen exposure may be involved in imprinting of the hypothalamus that could possibly influence pubertal onset many years later.[31]

We also found that children whose mothers had preeclampsia with severe features were drastically different from children whose mothers had a clinically mild or moderate preeclampsia. One possible explanatory mechanism that has been suggested, is that different steroid enzyme activity may differ between the groups.[11] Nonetheless, the interpretation of the heterogeneous findings is an obvious challenge, and the results warrant confirmation by others. However, our findings do suggest that intrauterine exposure to maternal preeclampsia is associated with pubertal development, and that timing and tempo of puberty may differ according to clinical features of preeclampsia.

Body mass and lifestyle factors appear to play an important role for maternal risk of relatively late-onset preeclampsia [32], which clinically tends to be relatively mild or moderate. [25,33,34] On the other hand, preeclampsia with severe features seems to be more strongly related to maternal chronic hypertension or to poor placentation, and long-term consequences for the offspring may therefore differ from those of the clinically mild cases. [35]

There is evidence that androgen excess leads to increased risk of cardiovascular disease later in life [36], and that women with a history of preeclampsia are at increased risk of heart disease.[37] In the context of our findings, it would be of particular interest to study cardiovascular risk in the offspring, by severity of preeclampsia. It has also been suggested that the pathway through which a girl enters puberty may have implications for her adult health, especially in relation to obesity and breast cancer risk later in life.[15] Thus, women with a history of preeclampsia seem to be at reduced risk for breast cancer [6,38–41], and it has been suggested that fetal exposure to androgens may lead to a life-time reduction in breast cancer risk.[9] Analogous to the situation among girls, it seems plausible that the androgen profile in boys may be relevant for future health. Thus, a delayed androgen activity may be associated with a reduced lifetime risk for prostate [42] and testicular cancer.[43,44]

Our findings suggest that maternal preeclampsia is associated with distinct hormonal milieus in the offspring, depending on whether the offspring was born without *in utero* exposure to preeclampsia, or with exposure to clinically mild, moderate or severe disease. The heterogeneous androgen patterns between preeclampsia subgroups, especially for total testosterone and DHEAS, may reflect different timing in pubertal development. It is not known whether these hormonal patterns persist into adult life or whether intrauterine exposure to preeclampsia has long-term clinical implications, but increased life-time risk of cardiovascular disease [37] and possibly reduced risk of breast cancer, seem plausible.[39]

## Supporting Information

S1 Table Offspring characteristics at follow-up according to preeclampsia status at delivery. (DOCX)

S2 Table Hormonal concentrations among mothers and offspring at 11–12 years by exposure to preeclampsia *in utero*. (DOCX)

S3 Table Testicular volume in boys at approximately 12 years by preeclampsia status. (DOCX)

S4 Table Hormonal differences^a^ from the reference group (no preeclampsia) among offspring at 11–12 years by exposure to preeclampsia *in utero*. (DOCX)

S1 Fig Box plots of testosterone, DHEAS, androstenedione and IGF-I concentrations in boys according to preeclampsia status. (DOCX)

S2 Fig Box plots of testosterone, DHEAS, androstenedione and IGF-I concentrations in girls according to preeclampsia status. (DOCX)

S1 Text Questionnaire English. (DOCX)

S2 Text Questionnaire Norwegian. (DOCX)
