# Supplementary material for: Maternal Preeclampsia and Androgens in the Offspring around Puberty: A Follow-Up Study
Source: PLoS One. 2016 Dec 19;11(12):e0167714. doi: 10.1371/journal.pone.0167714 (PMC5167253; doi:10.1371/journal.pone.0167714)
Supplement: S1 Table — (DOCX) [file pone.0167714.s001.docx]

SUPPLEMENTARY MATERIAL

Supplemental Table 1. Offspring characteristics at follow-up according to preeclampsia status at delivery^a^.

| Preeclampsia status | No  Mean±SD (SD)^d^ | Clinically mild  Mean±SD | | Clinically moderate  Mean±SD | | Severe features  Mean±SD | |  |
| --- | --- | --- | --- | --- | --- | --- | --- | --- |
| Girls |  |  |  |  |  |  |  |  |
| Age (years) | 10.8±0.1 | | 10.9±0.2 | | 10.8±0.1 | | 10.9±0.2 | |
| Height (cm) | 147.1±7.2 | | 147.0±7.3 | | 146.6±6.5 | | 147.4±7.7 | |
| Weight (kg) | 38.0±8.3 | | 40.1±10.3 | | 39.5±8.1 | | 40.7±10.3 | |
| BMI^b^ (kg/m^2^) | 17.5±2.8 | | 18.4±3.8 | | 18.4±2.9 | | 18.5±3.3 | |
| Waist circumference (cm) | 61.6±7.3 | | 65.0±9.7 | | 62.6±7.7 | | 64.6±7.8 | |
| Skinfold thickness, triceps (mm) | 11.6±4.3 | | 13.7±6.6 | | 13.1±4.8 | | 12.1±4.7 | |
| Tanner^c^ | 1.3±0.7 | | 1.2±0.4 | | 1.3±0.6 | | 1.4±0.7 | |
| Boys |  |  |  |  |  |  |  |  |
| Age (years) | 11.8±0.1 | 11.8±0.1 | | 11.8±0.1 | | 11.8±0.2 | |  |
| Height (cm) | 151.6±6.9 | 153.34±7.7 | | 153.9±6.0 | | 147.5±7.5 | |  |
| Weight (kg) | 41.5±8.3 | 44.3±8.6 | | 44.3±11.4 | | 38.6±7.6 | |  |
| BMI^b^ (kg/m^2^) | 18.1±2.7 | 18.8±2.9 | | 18.6±4.0 | | 17.6±2.7 | |  |
| Waist circumference (cm) | 64.5±8.3 | 66.3±7.1 | | 65.6±9.0 | | 64.3±7.1 | |  |
| Skinfold thickness, triceps (mm) mer) | 11.2±4.3 | 12.0±4.6 | | 11.2±5.2 | | 11.3±5.3 | |  |
| Tanner^c^ | 1.3±0.6 | 1.4±0.6 | | 1.6±0.7 | | 1.3±0.5 | |  |

^a^Previously published by Øglænd et al. (15)

^b^BMI = Body mass index

^c^Measured by nurse

^d^SD = Standard deviation
