# Supplementary material for: Maternal Preeclampsia and Androgens in the Offspring around Puberty: A Follow-Up Study
Source: PLoS One. 2016 Dec 19;11(12):e0167714. doi: 10.1371/journal.pone.0167714 (PMC5167253; doi:10.1371/journal.pone.0167714)
Supplement: S2 Table — (DOCX) [file pone.0167714.s002.docx]

Supplemental Table 2. Hormonal concentrations among mothers and offspring at 11-12 years by

exposure to preeclampsia *in utero*^a^

| Preeclampsia status  N | No  383 | Clinically mild  73 | Clinically moderate  91 | Severe features  54 | P-value |
| --- | --- | --- | --- | --- | --- |
| Girls |  |  |  |  |  |
| Testosterone Total (ng/dL) | 48.6±2.9 | 14.3±7.3 | 10.4±5.2 | 80.2±8.5 | <0.001 |
| DHEAS (ug/dL) | 23.7±1.9 | 57.7±4.7 | 52.5±3.4 | 8.8±5.5 | <0.001 |
| Androstenedione (ng/dL) | 49.9±3.0 | 53.8±7.6 | 47.3±5.4 | 44.8±8.8 | 0.95 |
| IGF-I (ng/mL) | 289.0±8.6 | 287.3±21.7 | 291.3±15.6 | 275.2±25.2 | 0.96 |
| Boys |  |  |  |  |  |
| Testosterone Total (ng/dL) | 62.7±7.5 | 102.3±15.9 | 88.7±18.6 | 84.0±18.5 | <0.001 |
| DHEAS ^b^ (ug/dL) | 58.8±2.3 | 66.9±4.8 | 69.8±5.6 | 5.8±5.6 | <0.001 |
| Androstenedione (ng/dL) | 32.5±1.1 | 33.5±2.4 | 31.8±2.8 | 30.1±2.8 | 0.18 |
| IGF-I ^c^ (ng/mL) | 258.2±6.9 | 292.9±14.6 | 295.8±17.1 | 223.7±17.1 | 0.004 |
| Mothers |  |  |  |  |  |
| Androstenedione (ng/dL) | 116.4±3.2 | 112.3± 7.0 | 107.2±6.5 | 118.4±8.9 | 0.39 |
| IGF-I (ng/mL) | 196.9±2.8 | 199.9±6.2 | 185.6±5.7 | 199.2±7.8 | 0.26 |
| SHBG^d^ (nmol/L) | 63.6±3.0 | 64.7±6.6 | 62.3. ±6.1 | 70.8±8.4 | 0.40 |

^a^ Adjusted for maternal age and education (least square means and standard errors)

^b^ DHEAS = Dehydroepiandrosterone sulfate

^c^ IGF-I = insulin-like growth factor 1

^d^ SHBG = Sex hormone binding globuline
