# Supplementary material for: Maternal Preeclampsia and Androgens in the Offspring around Puberty: A Follow-Up Study
Source: PLoS One. 2016 Dec 19;11(12):e0167714. doi: 10.1371/journal.pone.0167714 (PMC5167253; doi:10.1371/journal.pone.0167714)
Supplement: S3 Table — (DOCX) [file pone.0167714.s003.docx]

Supplemental Table 3. Testicular volume in boys at approximately 12 years by preeclampsia status^a^

| Preeclampsia status  N | No  184 | Clinically mild  43 | | Clinically moderate  30 | Severe features  29 | P-value |
| --- | --- | --- | --- | --- | --- | --- |
| Boys (unit) |  |  |  |  |  |  |
| Testicular volume (ml) | 5.15±0.23 | 6.22±0.45 | | 6.73±0.58 | 5.05±0.55 | 0.015 |

^a^ Adjusted for maternal age and education (least square means and standard errors)
