# Supplementary material for: Maternal Preeclampsia and Androgens in the Offspring around Puberty: A Follow-Up Study
Source: PLoS One. 2016 Dec 19;11(12):e0167714. doi: 10.1371/journal.pone.0167714 (PMC5167253; doi:10.1371/journal.pone.0167714)
Supplement: S4 Table — (DOCX) [file pone.0167714.s004.docx]

Supplemental table 4. Hormonal differences^a^ from the reference group (no preeclampsia) among offspring at 11-12 years by exposure to preeclampsia *in utero.*

| Preeclampsia status | Clinically mild and moderate  Difference (p-value)  ) |  | Severe features  Difference (p-value) |
| --- | --- | --- | --- |
| Girls (unit) |  |  |  |
| Testosterone Total (ng/dL) | -36.9 (<0.001) |  | 31.6 (<0.001) |
| DHEAS (ug/dL)^b^ | 30.5 (<0.001) |  | -14.9 (0.011) |
| Androstenedione (ng/dL) | -0.39 (0.792) |  | -5.10 (0.657) |
| IGF-I (ng/mL)^c^ | 0.86 (0.955) |  | -13.8 (0.604) |
| Boys (unit) |  |  |  |
| Testosterone Total (ng/dL) | 33.9 (0.001) |  | 21.4 (<0.001) |
| DHEAS ^c^ (ug/dL) | 9.29 (0.033) |  | -53.0 (<0.001) |
| Androstenedione (ng/dL) | 0.33 (0.443) |  | -2.36 (0.677) |
| IGF-I ^d^ (ng/mL) | 35.0 (0.007) |  | -34.4 (0.063) |
| Testicular volume (mL) | 1.256 (0.03) |  | -0.107 (0.858) |

^a^ Adjusted for maternal age and education (least square means)

^b^ DHEAS = Dehydroepiandrosterone sulfate

^c^ IGF-I = insulin-like growth factor 1
