# Supplementary material for: Maternal Preeclampsia and Androgens in the Offspring around Puberty: A Follow-Up Study
Source: PLoS One. 2016 Dec 19;11(12):e0167714. doi: 10.1371/journal.pone.0167714 (PMC5167253; doi:10.1371/journal.pone.0167714)

Supplemental Figure 1. Box plots of testosterone, DHEAS, androstenedione and IGF-I concentrations in boys according to preeclampsia status


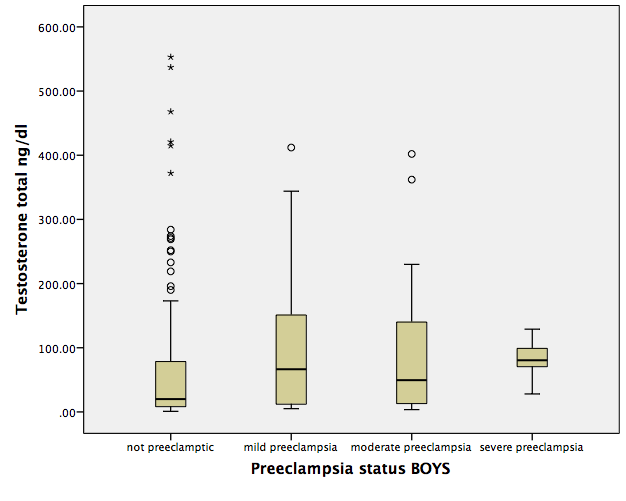

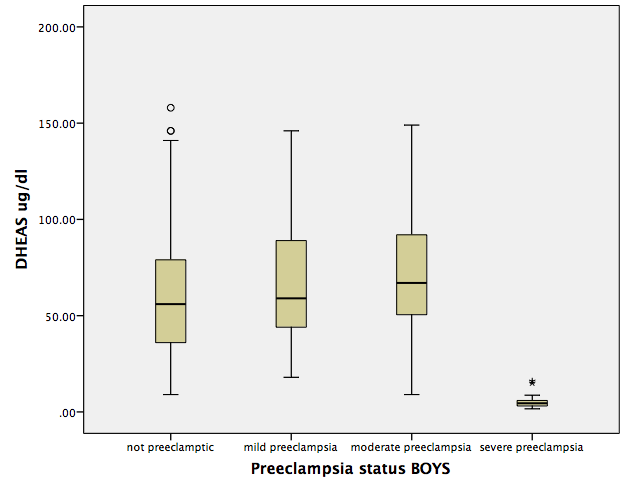


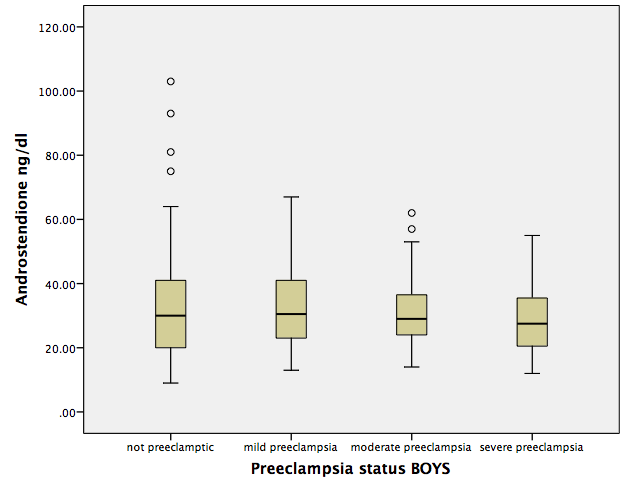

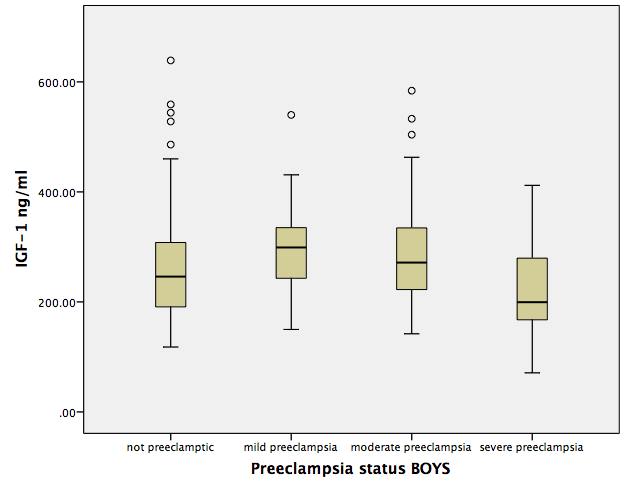

Supplement: S1 Fig — (DOCX) [file pone.0167714.s005.docx]
