# Supplementary material for: Maternal Preeclampsia and Androgens in the Offspring around Puberty: A Follow-Up Study
Source: PLoS One. 2016 Dec 19;11(12):e0167714. doi: 10.1371/journal.pone.0167714 (PMC5167253; doi:10.1371/journal.pone.0167714)
Supplement: S1 Text — (DOCX) [file pone.0167714.s007.docx]

Mother's Questionnaire

**________________________________________________________________**

**Section I: Your Pregnancy (Please fill in or check answer)**

All of the following questions relate to your pregnancy with *(Fill in with index child’s name*.

1. a) What was the **birth weight** of this child?

Don’t remember

grams

2 a) What was this child's **length** at birth?

Centimeters

(cm)

Don’t remember

3. a) What was the due date for this child?

Day

Month

Year

**1**

**9**

1. If you do not remember the exact due date, was the child born *(Check one box)*

More than 8 weeks early?

4 - 8 weeks early?

2 – 4 weeks early?

Less than 2 weeks before the due date?

On due date?

Less than 2 weeks past the due date?

More than 2 weeks past the due date?

Do not remember

4 What was your usual weight **before** you became pregnant with ________________?

*(Please give your best estimate.) (index child’s name)*

Kilograms

(kg)

5. Approximately, how much weight did you gain during this pregnancy? *(Check one box)*

Less than 5 kg  15 – 20 kg

5 – 7 kg  More than 20 kg

7 – 10 kg  Don’t remember

10 – 15 kg

6. a) Did you see a doctor or midwife during pregnancy?

Yes  No *(skip to question 7)*

b) When did you have your first check up during pregnancy? *(Check one box)*

During the first three months of pregnancy

After the first three months of pregnancy, but before 6 months

During the last three months of pregnancy

c) How was the baby delivered? *(Check one box)*

Vaginally  C-section

7. Was a forceps or vacuum used when you delivered this baby?

Yes  No  Don’t know

8. Did you have morning sickness (nausea) during this pregnancy?

Yes  No *(Skip to question #10)*

a) Which response best describes the nausea? *(Check only one box)*

Mild (no vomiting)

Moderate (some vomiting)

Severe (excessive vomiting)

b) When did you experience nausea? *(Check all that apply)*

During the first three months of pregnancy

During the second three months of pregnancy

During the last three months of pregnancy

9. During this pregnancy, did your physician prescribe any of the following medication(s)?

*(Please check Yes or No for each)*

a) Any medications for sleeping?  Yes  No

b) Any medications for nausea?  Yes  No

c) Cortisone?  Yes  No

10. At any time during your pregnancy with this child, did your physician or health care

provider diagnose you with any of the following conditions? *(Check Yes or No for each)*

a) Anemia………………………………………………..  Yes  No

**If Yes,** did you take iron supplements?…..  Yes  No

b) Diabetes………………………………………………  Yes  No

**If Yes,** did you take insulin?…………………….  Yes  No

c) High blood pressure…………………………………. Yes  No

d) Pre-eclampsia or eclampsia (toxemia)…………….  Yes  No

e) Proteinuria (protein in the urine)……………………  Yes  No

f) Infection (kidneys, respiratory, etc.)………………… Yes  No

11. a) During your pregnancy with ______________ *(index child’s name)*, did you take a

multiple or prenatal vitamin?

Yes  No

b) Did you take this vitamin regularly?

Yes  No

12. a) Were you ever hospitalized during your pregnancy with ________________ other

than the time of delivery? *(index child’s name)*

Yes  No

b) What trimester? *(Check all that apply)*

First trimester

Second trimester

Third trimester

c) For what reason were you hospitalized?

An illness related to the pregnancy

An illness unrelated to the pregnancy *(Please specify for each illness)*

_______________________________________________________  An injury (e.g., car accident)

13. When you were pregnant with ________________ *(index child’s name)*, how would you

classify your physical activity patterns at work (outside the home)? Would you say that they were: *(Check one box)*

Not applicable/Not working

Mostly sitting and standing

Mostly walking with some sitting and standing

Mostly heavy labor with some walking and standing and little sitting

14. When you were pregnant with ________________ *(index child’s name)*, how would you

classify your physical activity patterns **at home**? Would you say that they were:

*(Check one box)*

Mostly sitting

Mostly walking and standing with some sitting

Active housework most of the time with little sitting

Heavy manual work at home

15. When you were pregnant with ________________ *(index child’s name)*, aside from

housework and job-related activities, which of the following best describes your physical activity (e.g., walking and recreation)? *(Check one box)*

Highly active (equivalent to walking about 3 or more miles a day)

Somewhat active (equivalent to walking about 2 miles every day)

Active (equivalent to walking about 1 mile every day)

Mostly inactive (equivalent to walking about half a mile or less every day)

Inactive (no walking or other regular exercise)

16. During the second half of your pregnancy, did your overall physical activity:

*(Check one box)*

Stay about the same

Substantially increase

Substantially decrease

17. Did you ever smoke during your pregnancy with ________________?

*(index child’s name)*

Yes  No *(Skip to question #19)*

a) Please specify the number of cigarettes that you smoked per day.

[ 1 pack = 20 cigarettes] *(Check one box)*

1-14 cigarettes per day

15-24 cigarettes per day

25-34 cigarettes per day

35 or more cigarettes per day

1. Did you stop smoking during your pregnancy with this child?

Yes  No *(Skip to question #19)*

d) Please specify when. *(Check one box)*

During the first three months of pregnancy

During the second three months of pregnancy

During the last three months of pregnancy

18. At the time of this child’s birth, how many years of education had you completed?

*(Check one box)*

Less than 8 years of school  4 years of high school

8 years of school  1 - 3 years of college

1 – 3 years of high school  4 or more years of college

19. Did you own a home at the time of this child’s birth?

Yes  No

20. a) The following refers to your main work occupation **during your pregnancy** with this

child. Please mark the response which best describes your job at that time.

Teacher, librarian, medical doctor, lawyer

Nurse

Executive manager, administrator

Sales, clerical worker or secretary

Craftwork (e.g., seamstress, artist)

Machine operator, assembler, inspector, bus/cab driver

Service worker (e.g., housekeeper, janitor, waitress, equipment cleaner)

Laborer, handler

Farming

Homemaker

Did not work

b) The following refers to your main work occupation **during the infancy and**

**childhood of** this child. Please mark the response which best describes your job at that time.

Teacher, librarian, medical doctor, lawyer

Nurse

Executive manager, administrator

Sales, clerical worker or secretary

Craftwork (e.g., seamstress, artist)

Machine operator, assembler, inspector, bus/cab driver

Service worker (e.g., housekeeper, janitor, waitress, equipment cleaner)

Laborer, handler

Farming

Homemaker

Did not work

21. a) At the time of this child’s birth, were you living with his/her biological father or

a partner?

Yes  No *(Skip to question #23)*

1. At the time of this child’s birth, how many years of education had

he completed? *(Check one box)*

Less than 8 years of school  4 years of high school

8 years of school  1 - 3 years of college

1 – 3 years of high school  4 or more years of college

1. The following refers to his work history. Please mark the response which best describes his job at the time of this child’s infancy and childhood.

Lawyer, medical doctor, college professor, teacher

Executive, manager, administrator

Sales or clerical worker

Mechanic, electrician, repairer or craft worker (e.g., carpenter)

Machine operator, assembler, inspector, bus/cab driver

Service worker (e.g., janitor, guard)

Laborer, handler, equipment cleaner, helper

Farming

Military

Did not work

22. a) Did your child’s father/your partner ever smoke **cigarettes** during the pregnancy or

the early childhood of this child?

Yes  No *(Please answer #23d)*

b) Please specify when he smoked. *(Check one box)*

During your pregnancy with this child

During the early childhood of this child

Both

c) Please specify the number of cigarettes that he smoked per day.

*(Check one box)*

1-14 cigarettes per day

15-24 cigarettes per day

25-34 cigarettes per day

35 or more cigarettes per day

d) Did your child’s father/your partner smoke a pipe and/or cigars daily?

Yes  No

**Section II**: **Diet during pregnancy.**

This section asks you about your drinking habits during pregnancy.

23. How much coffee did you drink daily during this pregnancy? (*do not include caffein-free coffee*)

Did not drink coffee

Less than one cup daily

1 – 2 cups daily

3 – 4 cups daily

5 or more daily

Don’t remember

24. How much tea did you drink daily during this pregnancy? (do not include herbal tea or caffein-free tea)

Did not drink tea

Less than one cup daily

1 – 2 cups daily

3 – 4 cups daily

5 cups or more daily

Don’t remember

25. How often did you drink alcholic beverages during this pregnancy? One unit of alcohol is equivalent to one bottle of cider, one glass (1/3 liter) beer, one glass of red or white wine, one serving of sherry, liquor or similar.

Did not drink alcohol when I was pregnant

Less than one unit per week

1 – 2 units of alcohol per week

3 – 6 units of alcohol per week

1 unit of alcohol per week

2 –3 units of alcohol per week

4 units of alcohol or more per day

Don’t remember

## Section III: Your Child’s Infancy

This section asks you about this child’s infancy.

26. If you had a girl, did you ever notice blood in the diaper during her first days of life?

Yes  No  I had a boy

27. Did you notice if there was a white discharge or fluid from the child’s nipples about one month after birth?

Yes  No

28. a) Did your child have inflammation (red swelling) in the breasts early in infancy?

Yes  No *(Skip to question 29)*

b) Was the treatment surgical?  Yes  No

29. Did this child ever have enlarged breasts before he/she was two years old?

Yes  No

30. a) Did you ever breast-feed this child?

Yes  No

b) **If yes**, at what age did you stop? *(Check one box)*

Less than one week  6 – 9 months

1 week – 3 months  9 – 12 months

3 – 6 months  One year or more

31. a) Did you feed this child **commercial infant formula (e.g., Similac, Enfamil,**

**SMA, etc.)** on a daily basis?

Yes  No

b) At what age did you start to give this to your child?

Before 3 months of age  6 – 9 months of age

3 – 6 months of age  9 months of age or older

c) At what age did you stop? *(Check one box)*

Less than one week  6 – 9 months

1 week – 3 months  9 – 12 months

3 – 6 months  One year or more

32. a) Did you ever feed this child a **soy-based infant formula** on a daily basis?

Yes  No

1. At what age did you start to give this to your child? *(Check one box)*

Before 3 months of age  6 – 9 months of age

3 – 6 months of age  9 months of age or older

1. At what age did you stop? *(Check one box)*

Less than one week  6 – 9 months

1 week – 3 months  9 – 12 months

3 – 6 months  One year or more

33. Did you ever feed your child Nutramigen on a daily basis?

a)

Yes  No

1. At what age did you start to give this to your child? *(Check one box)*

Before 3 months of age  6 – 9 months of age

3 – 6 months of age  9 months of age or older

1. At what age did you stop? *(Check one box)*

Less than one week  6 – 9 months

1 week – 3 months  9 – 12 months

3 – 6 months  One year or more

34. At what age did you start feeding this child **regular** **cow’s milk** (e.g., bottle or carton)?

*(Check one box)*

Never gave cow’s milk  6 – 9 months of age

Before 3 months of age  9 months of age or older

3 – 6 months of age

35. At what age did you start feeding this child **solid food**? *(Check one box)*

Before 3 months of age  6 - 9 months of age

3 – 6 months of age  9 months of age or older

36. What was this child’s first **solid** food? *(Check one box)*

Cereals

Fruits

Vegetables

Breads, crackers or cookies

## Section IV: Your Child’s Preschool Diet

This section asks you about this child’s preschool diet. Please try to remember when your child was of **preschool age (3 to 5 years)**. For each question, indicate how often this child ate or drank an average serving of the following item(s). Remember to include foods/ingredients that you cooked with. These questions are not intended to describe all of the foods eaten.

37. a) Identify which **type of milk** this child consumed **the most.**

*[Check one box]*

Whole cow’s milk  Soy milk

Low-fat cow’s milk  Goat’s milk

Skim cow’s milk

1. How frequently did ________________ drink this type of milk? *[Check one box]*

*(index child’s name)*

Never  1 glass per day

1 – 3 glasses per month  2 – 3 glasses per day

1 – 4 glasses per week  4 or more glasses per day

5 – 6 glasses per week

38. **Ice Cream** *[Check one box]*

Never  2 – 4 times per week

1 – 3 times per month  5 or more times per week

Once a week

39. **Cheese** *[Check one box]*

Never  1 pat per day

1 – 3 pats per month  2 – 4 pats per day

1 pat per week  5 or more pats per day

2 – 6 pats per week

40. **Margarine** (oleo) *[Check one box]*

Never  1 pat per day

1 – 3 pats per month  2 – 4 pats per day

1 pat per week  5 or more pats per day

2 – 6 pats per week

41. **Butter** *[Check one box]*

Never  1 pat per day

1 – 3 pats per month  2 – 4 pats per day

1 pat per week  5 or more pats per day

2 – 6 pats per week

42. **Peanut butter** *[Check one box]*

Never  2 – 4 times per week

1 – 3 times per month  5 or more times per week

Once a week

43. **Mayonnaise** *[Check one box]*

Never  2 – 4 times per week

1 – 3 times per month  5 or more times per week

Once a week

44. **Apples** *[Check one box]*

Never  2 – 4 times per week

1 – 3 times per month  5 or more times per week

Once a week  1 or more times per day

45. **Bananas** *[Check one box]*

Never  2 – 4 times per week

1 – 3 times per month  5 or more times per week

Once a week  1 or more times per day

46. **Raisins** *[Check one box]*

Never  2 – 4 times per week

1 – 3 times per month  5 or more times per week

Once a week  1 or more times per day

47. **Oranges** *[Check one box]*

Never  2 – 4 times per week

1 – 3 times per month  5 or more times per week

Once a week  1 or more times per day

48. **Orange juice** *[Check one box]*

Never  1 glass daily

1 – 3 glasses per month  2-3 glasses daily

1-4 glasses per week  4 glasses or more daily

5-6 glasses per week

49. **Apple juice** *[Check one box]*

Never  1 glass daily

1 – 3 glasses per month  2-3 glasses daily

1-4 glasses per week  4 glasses or more daily

5-6 glasses per week

50. **Broccoli** *[Check one box]*

Never  2 – 4 times per week

1 – 3 times per month  5 or more times per week

Once a week

51. **Carrots** *[Check one box]*

Never  2 – 4 times per week

1 – 3 times per month  5 or more times per week

Once a week

52. **Green beans** *[Check one box]*

Never  2 – 4 times per week

1 – 3 times per month  5 or more times per week

Once a week

53. **Peas** *[Check one box]*

Never  2 – 4 times per week

1 – 3 times per month  5 or more times per week

Once a week

54. **Corn** *[Check one box]*

Never  2 – 4 times per week

1 – 3 times per month  5 or more times per week

Once a week

55. **Spinach** *[Check one box]*

Never  2 – 4 times per week

1 – 3 times per month  5 or more times per week

Once a week

56. **Eggs** *[Check one box]*

Never  2 – 4 eggs per week

1 – 3 eggs per month  5 eggs or more per week

One egg per week

1. **Hot dogs** *[Check one box]*

Never  2 – 4 times per week

1 – 3 times per month  5 or more times per week

Once a week

1. **Sliced meats (sandwich meats)** *[Check one box]*

Never  2 – 4 times per week

1 – 3 times per month  5 or more times per week

Once a week

1. **Ground beef** (e.g., hamburgers, meatloaf) *[Check one box]*

Never  2 – 4 times per week

1 – 3 times per month  5 or more times per week

Once a week

60. **Beef, pork or lamb** (e.g., roast, steaks, stews) *[Check one box]*

Never  2 – 4 times per week

1 – 3 times per month  5 or more times per week

Once a week

61. **Chicken or turkey** *[Check one box]*

Never  2 – 4 times per week

1 – 3 times per month  5 or more times per week

Once a week

62. **Fish/ seafood** (e.g., fresh or canned, fried, baked, broiled) *[Check one box]*

Never  2 – 4 times per week

1 – 3 times per month  5 or more times per week

Once a week

63. **Liver** *[Check one box]*

Never  2 – 4 times per week

1 – 3 times per month  5 or more times per week

Once a week

64. **Tomato or spaghetti sauce** *[Check one box]*

Never  2 – 4 times per week

1 – 3 times per month  5 or more times per week

Once a week

65. **Pizza** *[Check one box]*

Never  2 – 4 times per week

1 – 3 times per month  5 or more times per week

Once a week

66. **Pasta** *[Check one box]*

Never  2 – 4 times per week

1 – 3 times per month  5 or more times per week

Once a week

67. **Bread** *[Check one box]*

Never  5 – 7 slices per week

1 slice per week or less  2 – 3 slices per day

2 – 4 slices per week  4 slices per day or more

68. **Bakery products** (e.g., donuts, cookies, muffins, crackers, cakes, rolls, pastries, pies)

*[Check one box]*

Never  2 – 4 times per week

1 – 3 times per month  5 or more times per week

Once a week

69. **Rice** *[Check one box]*

Never  2 – 4 times per week

1 – 3 times per month  5 or more times per week

Once a week

70. **Breakfast cereal** *[Check one box]*

Never  2 – 4 bowls per week

1 – 3 bowls per month  5 – 7 bowls per week

1 bowl per week  2 or more bowls per day

71. **Potatoes** (baked, boiled, mashed - **NOT FRIED**) *[Check one box]*

Never  2 – 4 times per week

1 – 3 times per month  5 or more times per week

Once a week

72. a) Did the child eat fried potatoes or french fries while in preschool?

Yes  No

1. How often did the child eat french fries or fried potatoes? *(Check one box)*

A few times

1 – 3 times per month  2 – 4 times per week

Once a week  5 or more times per week

1. How were the potatoes prepared: *(Check one box)*

From frozen condition, in the frying pan or in the oven

Fast food (from McDonald’s or similar)

Fried at home *(If yes, answer question 72d)*

1. If you prepared french fries at home, what kind of fat did you use? *(Check one box)*

Butter  Plantbased oil

Chickenfat  Plantbased margarine

73. How often did the child eat other kinds of fried potatoes (for example potato chips)? *(Check one box)*

Never  2 – 4 times per week

1 – 3 times per month  5 or more times per week

Once a week

74. What **type of fat** did you use most often to fry foods at home (e.g., fish, chicken)?

*[Check all that apply]*

Butter  Vegetable oil

Margarine (oleo)  Corn oil

Vegetable shortening  Peanut oil

(e.g., Crisco)

Lard  Safflower oil

Bacon grease  Olive oil

Chicken fat  None

75. When ________________ *(index child’s name)* was a preschooler, did you give him/her multiple vitamins? *(Check one box)*

Never  2 – 4 times per week

1 – 3 times per month  5 or more times per week

Once a week

76. When ________________ *(index child’s name)* was a preschooler, did you give him/her cod liver oil? *(Check one box)*

Never  2 – 4 times per week

1 – 3 times per month  5 or more times per week

Once a week

**Section V: Physical Activity of the index child, History of illnesses, and Medications**

Now please answer questions regarding this child’s activity.

77. Think about this child’s physical activity between the ages of 3 and 6 years. How would

you best describe your child’s activity level compared to other children of the same age/sex? Was your child: *(Check one box)*

A lot more physically active than most

Average – same as most

A little less physically active than most

A lot less physically active than most

78. Between the ages of 3 and 6 years, how many hours per day did the child watch television? (*Check one box*)

Did not watch TV  About 3 hours per day

< 30 minutes per day  About 4 hours per day

About 1 hour per day  5 hours or more per day

About 2 hours per day

79. Think about your child’s physical activity after the age of 6 years. How would

you best describe your child’s activity level compared to other child of the same age/sex? Was your child: *(Check one box)*

A lot more physically active than most

Average – same as most

A little less physically active than most

A lot less physically active than most

80. Between the ages of 6 and 10 years, how many hours per day did the child watch television? (*Check one box*)

Did not watch TV  About 3 hours per day

< 30 minutes per day  About 4 hours per day

About 1 hour per day  5 hours or more per day

About 2 hours per day

81. Has ________________ *(index child’s name)* ever had any of the following illnesses?

*(Please check YES or NO for each)*

a) Diabetes………………………………………………...  Yes  No

b) Pneumonia……………………………………………..  Yes  No

c) Kidney infection………………………………………..  Yes  No

d) Asthma (doctor diagnosed)…………………………..  Yes  No

e) Allergies – respiratory…………………………………  Yes  No

f) Allergies – skin………………………………………….  Yes  No

g) Hospitalization, *specify*______________________  Yes  No

h) Cancer, *specify*____________________________  Yes  No

i) Tonsils removed………………………………………..  Yes  No

j) Bacterial infection, *specify*____________________  Yes  No

82. a) Is ________________ *(index child’s name)* on any medications now?

Yes  No

b) *If so, please specify* ____________________________________

## Section VI: Some Questions About Yourself

83. What is your date of birth?

Month

Day

Year

**1**

**9**

84. What was your birth weight in kilograms (kgs)? **NEED CATEGORIES FOR KG**

Less than 2.5 kg  > 4.5 kg

2.5 – 3.9 kg  Don’t know

4 – 4.5 kg

85. Were you born in Norway?

Yes  No

86. What is your marital status? *(Check one box)*

Married/cohabitant  Separated  Divorced

Widowed  Never been married or in a domestic partnership

87. How tall were you at about age 20 – 30?

Centimeters (cm)

88. If you gain weight, where on the body does it show best? (*Check the appropriate boxes*)

Around chest and shoulders

Around the stomach

Around hips/thighs

All over

Other *(Specify)* ________________________________

I never gain weight

89. Which race(s) do you consider yourself to be? *(Check all that apply)*

Caucasian/White (non-Hispanic/Latina)  Asian/Pacific Islander

African American/Black (non-Hispanic/Latina)

Hispanic or Latina  Other *(Specify):*_________________

## REPRODUCTIVE HISTORY

We are interested in your reproductive history, including your age at menarche, and your current menstrual history (if you are still menstruating).

90. At what age did you experience your first menstrual period? *(Check one box)*

Less than 11 y  14 y  18 y or later

11 y  15 y  Don’t know

12 y  16 y

13 y  17 y

91. What is the usual length of your menstrual cycle (interval from the first day of your period to the first day of your next period)? *(Check one box)*

Less than 21 days  40 or more days

21 – 25 days  Too irregular to estimate

26 – 31 days  Had no periods

32 – 39 days

92. a) The following table relates to your childbirth history. Include all pregnancies

lasting 6 months or longer. Include ________________ *(index child’s name)*, as well

as twins. Answer one line apiece for each twin.

| **Date of Birth**  **(MM/DD/YY)** | Outcome | Gender |
| --- | --- | --- |
|  | Stillbirth  Live birth | M  F |
|  | Stillbirth  Live birth | M  F |
|  | Stillbirth  Live birth | M  F |
|  | Stillbirth  Live birth | M  F |
|  | Stillbirth  Live birth | M  F |
|  | Stillbirth  Live birth | M  F |
|  | Stillbirth  Live birth | M  F |
|  | Stillbirth  Live birth | M  F |
|  | Stillbirth  Live birth | M  F |
|  | Stillbirth  Live birth | M  F |
|  | Stillbirth  Live birth | M  F |
|  | Stillbirth  Live birth | M  F |

1. Did you ever have a miscarriage?

Yes  No

How many?  1  2  3  4  5 or more

93. Have you ever tried to become pregnant for more than one year

without success? *(Check one box)*

Yes  No

94. Have you or your partner used any form of birth control during the last 12 months?

Yes  No

Would rather not answer

*(skip to question 96)*

95. Check the boxes that apply for the last 12 months.

Birth control pill

Birth control vaginal ring

Birth control shot

Birth control implant

Condom

Spermicide

Female condom

Intrauterine device (IUD) (copper)

Intrauterine device (IUD) (hormonal)

Sterilization (woman)

Sterilization (man)

Fertility awareness based methods (“safe” periods)

Withdrawal

Other methods *(Specify)* ____________________________

96. Are you pregnant now?

Yes

No

97. Have you had a hysterectomy ?

Yes

No

98. a) Were you ever diagnosed with **hypertension** (high blood pressure) by a physician,

**when you were not pregnant**,?

Yes  No

b) At what age were you diagnosed? ________________years

99. a) Have you ever been diagnosed with endometriosis?

Yes  No

b) At what age were you diagnosed? ________________years

100. a) Have you ever been diagnosed with polycystic ovaries (PCO)?

Yes  No

b) At what age were you diagnosed? ________________years

101. a) Have you ever been diagnosed with breast cancer?

Yes  No

b) At what age were you diagnosed? ________________years

102. Did your biological mother have breast cancer?

Yes  No  Don’t Know

103. a) Was your biological mother ever diagnosed with preeclampsia?

Yes  No  Don’t know

b) When she was pregnant with you?

Yes  No

c) When she was pregnant with my

brother

sister

104. a) Do you have any **biological sisters**? (Include deceased; do not include half

siblings)

Yes  No (*skip to question 105*)

1. How many?

1  3

2  Four or more

1. Were any of them diagnosed with breast cancer?

Yes  No  Don’t Know

d) Her age at cancer diagnosis:

Sister’s age  Less than 45  50 – 55

45 – 49  56 or older

Sister’s age  Less than 45  50 – 55

45 – 49  56 or older

Sister’s age  Less than 45  50 – 55

45 – 49  56 or older

e) Were any of them diagnosed with preeclampsia?

Yes  No *(Skip to question 105)*

f) How many times?  1  2  3  4 or more

105. Have you ever had any of the following illnesses? *(Check Yes or No for each)*

a) Diabetes mellitus…………………………………………. Yes  No

b) Osteoporosis……………………………………………...  Yes  No

c) Hip Fracture after 60...…………………………………...  Yes  No

d) Myocardial infarction (MI) (heart attack)………………..  Yes  No

e) Asthma (doctor diagnosed)……………………………...  Yes  No

f) Fibrocystic/Other benign breast disease……………….  Yes  No

g) Cancer of the cervix………………………………………  Yes  No

h) Cancer of the uterus (endometrium)……………………  Yes  No

i) Cancer of the ovary………………………………………  Yes  No

j) Colon or rectal polyp……………………………………..  Yes  No

k) Cancer of the colon or rectum…………………………..  Yes  No

l) Cancer of the lung………………………………………..  Yes  No

m) Other cancer………………………………………………  Yes  No

106. a) Do you currently use any kind of medication?

Yes  No

1. If yes, please specify:

_________________________________________

107. Did you fill out this questionnaire: *(Check all that apply)*

On your own from memory

By referring to a source (e.g., birth certificate or baby book)

With help from your child or someone else (e.g. spouse, relative, friend)

Thank you very much for your participation!

Please return this questionnaire in the enclosed, prepaid envelope.
