# Supplementary material for: Maternal Preeclampsia and Androgens in the Offspring around Puberty: A Follow-Up Study
Source: PLoS One. 2016 Dec 19;11(12):e0167714. doi: 10.1371/journal.pone.0167714 (PMC5167253; doi:10.1371/journal.pone.0167714)
Supplement: S2 Text — (DOCX) [file pone.0167714.s008.docx]

Spørreskjema til mor

**______________________________________________________________**

**Del 1: Om svangerskapet ditt (Fyll inn eller kryss av for rett svar)**

1. Hva var barnets fødselsvekt?

Husker ikke

gram

2 Hva var barnets fødselslengde?

Centimeter

Husker ikke

3. a) Hva var datoen for termin til dette barnet?

Dag

Måned

År

**1**

**9**

1. Dersom du ikke husker nøyaktig dato for termin, ble barnet født

*(Kryss av bare en gang)*

Mer enn 8 uker for tidlig?

4 - 8 uker for tidlig?

2 – 4 uker for tidlig?

Mindre enn 2 uker før termin?

Ved termin

Mindre enn to uker over tiden

Mer enn to uker over tiden?

Husker ikke når jeg hadde termin

1. Hva var din vanlige vekt før du ble gravid med dette barnet ?

*(Vær snill å gi et anslag dersom du er usikker.)*

Kg

1. Omtrent hvor mye la du på deg i løpet av svangerskapet? *(Kryss av en gang)*

Mindre enn 5 kg  15 – 20 kg

5 – 7 kg  Mer enn 20 kg

7 – 10 kg  Husker ikke

10 – 15 kg

6. a) Gikk du til svangerskapsundersøkelser hos lege / jordmor?

Ja  Nei *(gå til spørsmål 7)*

b) Når gikk du til svangerskapsundersøkelse første gang? *(Kryss en gang)*

I løpet av de første tre månedene av svangerskapet

Etter de tre første, men før jeg var seks måneder på vei

I løpet av de siste tre månedene av svangerskapet

c) Ble barnet forløst på vanlig måte eller med keisersnitt? *(Kryss en gang)*

På vanlig måte  Keisersnitt

7. Ble det brukt tang eller vakuum da du fødte dette barnet ?

Ja  Nei  Vet ikke

8. Hadde du morgenkvalme i dette svangerskapet ?

Ja  Nei *(Gå til spørsmål 9)*

a) Hvordan vil du beskrive kvalmen? *(Sett ett kryss)*

Mild (ikke oppkast)

Moderat (noe oppkast)

Alvorlig (mye oppkast)

b) Når i svangerskapet hadde du morgenkvalme? *(Ett eller flere kryss )*

I de første tre månedene

I de neste tre månedene

I de siste tre månedene

c) Oppsøkte du lege eller jordmor på grunn av kvalmen?

Ja  Nei

9. I løpet av dette svangerskapet, fikk du noen av følgende typer medikamenter av din lege? *(Kryss ja eller nei for hver linje)*

a) Sovemedisin?  Ja  Nei

b) Medisin mot kvalme?  Ja  Nei

c) Kortison i tablettform  Ja  Nei

(f.eks. Prednisolon tabletter)

10. Ble noen av diagnosene nedenfor stilt av din lege eller din jordmor i løpet

av dette svangerskapet? *(Kryss ja eller nei på hver linje)*

a) Anemi (jernmangel)…………………………………  Ja  Nei

Tok du jerntilskudd i dette svangerskapet ?…  Ja  Nei

b) Diabetes………………………………………………  Ja  Nei

**Hvis ja,** tok du insulin?…………………….  Ja  Nei

c) Høyt blodtrykk………………………………….  Ja  Nei

d) Svangerskapsforgiftning (preeklampsi)……………. Ja  Nei

e) Protein i urinen…………………………………  Ja  Nei

f) Infeksjon (nyrene, luftveiene)…………………  Ja  Nei

11. a) Tok du vitamintilskudd I løpet av dette svangerskapet?

Ja  Nei

b) Tok du vitaminer regelmessig (hver dag)?

Ja  Nei

1. a) Var du noen gang innlagt på sykehus I løpet av svangerskapet – unntatt når du skulle føde?

Ja  Nei

b) Når i svangerskapet? *(Kryss av der det passer)*

Første tre måneder

Neste tre måneder

Tre siste måneder

c) Hva var hovedgrunnen til innleggelsen?

Sykdom som hadde med svangerskapet å gjøre

Sykdom som ikke hadde noe med svangerskapet å gjøre

*(Vær snill å spesifisere)*

__________________________________________________  Skade (for eks. bilulykke)

13. Hvordan vil du beskrive din fysiske aktivitet utenom hjemmet (på arbeid) i løpet av dette svangerskapet? *(Kryss en gang)*

Mest sitting og ståing

Mest gåing, og noe sitting og ståing

Mest tungt arbeid med en del gåing og ståing, men lite sitting

*Spørsmålet passer ikke for min situasjon*

14. Hvordan vil du beskrive din fysiske aktivitet hjemme i dette svangerskapet ?

*(Sett ett kryss)*

Mest sitting

Mest gåing og ståing, og noe sitting

For det meste aktivt husarbeid med lite sitting

Tungt kroppsarbeid hjemme

15. Hvordan vil du beskrive din fysiske aktivitet i dette svangerskapet når du verken var hjemme eller på arbeid? *(Sett ett kryss)*

Svært aktiv (tilsvarer å gå minst 5 kilometer hver dag)

Nokså aktiv (tilsvarer å gå opptil 3 kilometer hver dag)

Aktiv (tilsvarer å gå mellom 1 og 2 kilometer hver dag)

For det meste lite aktiv (tilsvarer å gå mindre enn 1 kilometer hver dag)

Lite aktiv (ikke regelmessig gåing eller fysisk trening)

16. Hvordan vil du beskrive din fysiske aktivitet i andre halvdel av svangerskapet ?

*(Kryss en gang)*

Omtrent som i første halvdel

Betydelig økt

Betydelig redusert

17. Røykte du noen gang i dette svangerskapet ?

Ja  Nei *(Gå til spørsmål 18)*

a) Vær snill å krysse av antallet sigaretter som du røykte daglig.

[ 1 pakke = 20 sigaretter] *(Sett ett kryss)*

1-4 sigaretter daglig

5-9 sigaretter daglig

10-14 sigaretter daglig

15-24 sigaretter daglig

25 eller flere sigaretter daglig

1. Sluttet du å røyke i dette svangerskapet?

Ja  Nei *(Gå til spørsmål 18)*

d) Når i svangerskapet sluttet du? *(Sett ett kryss)*

I løpet av de tre første månedene

I løpet av de neste tre månedene

I løpet av de siste tre månedene

18. Hvor mange års utdanning hadde du da dette barnet ble født ?

*(Sett ett kryss)*

Mindre enn 9 års skolegang  Videregående (3 år)

9 års skolegang  1 - 3 år etter videregående

1 – 2 år videregående  4 år eller mer etter videregående

19. Eide du/dere et sted å bo da barnet ble født?

Ja  Nei

20. a) Vi vil gjerne vite hva slags arbeid du hadde i dette svangerskapet. Kryss av det som passer best.

lærer, bibliotekar, lege, advokat

sykepleier

daglig leder, administrator

ekspeditør I butikk, sekretær

fagarbeider

maskinoperatør, sjåfør

husholder, vaktmester, kelner, renholdsarbeider

ufaglært arbeider

bonde, landbruksarbeider

husmor

student/skoleelev

hadde ikke arbeid

b) Vi vil også vite om du hadde noe arbeid i småbarnsperioden. Vær snill å krysse av det som passer best.

lærer, bibliotekar, lege, advokat

sykepleier

daglig leder, administrator

ekspeditør I butikk, sekretær

fagarbeider

maskinoperatør, sjåfør

husholder, vaktmester, kelner, renholdsarbeider

ufaglært arbeider

bonde, landbruksarbeider

husmor

hadde ikke arbeid

21. a) Bodde du sammen med barnets biologiske far da barnet ble født ?

Ja  Nei *(Gå til spørsmål 22)*

1. Hvor mange års skolegang hadde barnets far da barnet ble født?

*(Kryss ett sted)*

Mindre enn 9 års skolegang  3 års videregående

9 år  1 - 3 år etter videregående

1 – 2 års videregående  4 år eller mer etter videregående

1. Hva slags arbeid hadde barnets far de første årene etter fødselen. Kryss av for det alternativet som passer best. ved fødselen og de første årene etter fødselen. Kryss av det alternativet som passer best.

lærer, bibliotekar, lege, advokat

sykepleier

daglig leder, administrator

ekspeditør I butikk, sekretær

fagarbeider

maskinoperatør, sjåfør

husholder, vaktmester, kelner, renholdsarbeider

ufaglært arbeider

bonde, landbruksarbeider

husmor

hadde ikke arbeid

22. a) Pleide barnets far å røyke da du var gravid – eller da barnet var nyfødt?

Ja  Nei *(Gå til 22d)*

b) Spesifiser når han røykte. *(Kryss ett sted)*

Da du var gravid

Da barnet var nyfødt

Begge deler

c) Omtrent hvor mye røykte han daglig?.

*(Kryss ett sted)*

1-14 sigaretter daglig

15-24 sigaretter daglig

25-34 sigaretter daglig

35 sigaretter eller mer daglig

d) Pleide barnets far/din samboer å røyke pipe eller sigar daglig?

Ja  Nei

## Del 2: Ditt kosthold i svangerskapet

I denne delen spør vi hva du pleide å drikke da du var gravid. For hvert spørsmål vil vi be deg å krysse av hvor mye du drakk av de følgende drikkevarene. Prøv å gi et gjennomsnitt for hele svangerskapet.

23. Hvor mye kaffe drakk du daglig i dette svangerskapet? *(Ikke ta med koffeinfri kaffe)*

Drakk ikke kaffe

Mindre enn en kopp daglig

1 – 2 kopper daglig

3 – 4 kopper daglig

5 kopper eller mer daglig

Husker ikke

24. Hvor mye te drakk du daglig i dette svangerskapet? *(Ikke ta med urtete eller te uten koffein)*

Drakk ikke te

Mindre enn en kopp daglig

1 – 2 kopper daglig

3 – 4 kopper daglig

5 kopper eller mer daglig

Husker ikke

25. Hvor ofte drakk du alkoholholdige drikkevarer i dette svangerskapet?

For å sammenligne ulike typer alkohol spør vi etter det vi kaller alkoholenheter. En alkoholenhet tilsvarer:

1 flaske rusbrus/cider,

1 glass (1/3 liter) øl,

1 vinglass rød eller hvitvin,

1 hetvinsglass sherry eller annen hetvin,

1 drammeglass brennevin eller likør

Drakk ikke alkohol da jeg var gravid

Mindre enn en alkoholenhet i uken

1 – 2 alkoholenheter i uken

3 – 6 alkoholenheter i uken

1 alkoholenhet daglig

2 –3 alkoholenheter daglig

4 alkoholenheter eller mer daglig

Husker ikke

## Del 3: Om barnet i spedbarnsperioden

I denne delen spør vi om barnet i spedbarnsperioden – dvs i første leveår.

26. Dersom du fikk en jente, la du noen gang merke til om det var friskt blod i bleien (”spedbarns-mens”) de første levedagene?

Ja  Nei  Jeg fikk en gutt

27. Hos mange babyer kommer det hvit væske fra brystvortene de første dagene etter fødselen. La du merke til om det kom hvit væske ut av brystvortene til barnet omtrent en måned etter fødselen?

Ja  Nei

28. a) Hadde barnet noen gang betennelse i brystet (rød hevelse) tidlig i spedbarnsperioden?

Ja  Nei *(Gå til spørsmål 29)*

b) Måtte betennelsen fjernes kirurgisk?  Ja  Nei

29. La du merke til om barnets bryster var unormalt store før to års alder?

Ja  Nei

30. a) Ammet du barnet?

Ja  Nei *(Gå til spørsmål 31)*

b) **Hvis ja**, hvor lenge ammet du? *(Kryss ett sted)*

Mindre enn en uke  6 – 9 måneder

1 uke – 3 måneder  9 – 12 måneder

3 – 6 måneder  Ett år eller mer

31. a) Fikk barnet fabrikkfremstilt morsmelkerstatning (f.eks. Nan, Collett e.l.) daglig?

Ja  Nei *(Gå til spørsmål 33)*

b) Når begynte du å gi barnet dette?

Før 3 måneders alder  6 – 9 måneder

3 – 6 måneders alder  9 måneder eller eldre

c) Når sluttet du? *(Kryss ett sted)*

Før barnet var en uke  6 – 9 måneder

1 uke – 3 måneder  9 – 12 måneder

3 – 6 måneder  Ett år eller mer

32. a) Fikk barnet soya-melk (Soya-semp) daglig?

Ja  Nei *(Gå til spørsmål 34)*

a) Når begynte du å gi barnet dette? *(Kryss ett sted)*

Før 3 måneders alder  6 – 9 måneder

3 – 6 måneders alder  9 måneder eller eldre

b) Når sluttet du? *(Kryss ett sted)*

Før barnet var en uke  6 – 9 måneder

1 uke – 3 måneder  9 – 12 måneder

3 – 6 måneder  Ett år eller mer

33. Fikk barnet Nutramigen daglig?

a)

Ja  Nei

1. Når begynte du å gi dette til barnet Nutramigen? *(Kryss ett sted)*

Før 3 måneders alder  6 – 9 måneder

3 – 6 måneder  9 måneder eller eldre

1. Når sluttet du å gi dette til barnet ? *(Kryss ett sted)*

Før barnet var en uke  6 – 9 måneder

1 uke – 3 måneders alder  9 – 12 måneder

3 – 6 måneder  Ett år eller mer

34. Når begynte du å gi barnet vanlig kumelk? *(Kryss ett sted)*

Fikk ikke kumelk  6 – 9 måneder

Før 3 måneders alder  9 måneder eller eldre

3 – 6 måneder

35. Når begynte du å gi barnet fast føde? *(Kryss ett sted)*

Før 3 måneders alder  6 - 9 måneder

3 – 6 måneder  9 måneder elle eldre

36. Hva var den første faste maten du begynte med? *(Kryss ett sted)*

Grøt

Frukt

Grønnsaker / potet

Brød, kjeks

## Del 4: Kostholdet til barnet før skolealder

I denne delen spør vi om maten barnet spiste i førskolealder (3-6 år). Prøv å tenke godt etter, og kryss av hvor ofte barnet spiste følgende matvarer. Det er ikke meningen å spørre om alt barnet spiste.

37. a) Hva slags melk drakk barnet for det meste ?

*[Kryss ett sted]*

Helmelk  Skummet melk

Lettmelk  annen melk (soyamelk, geitemelk)

*P*

1. Hvor ofte drakk barnet melk ? *[Kryss ett sted]*

Aldri  1 glass daglig

1 – 3 glass i måneden  2 – 3 glass daglig

1 – 4 glass i uken  minst 4 glass daglig

5 – 6 glass i uken

38. **Iskrem** *[Kryss ett sted]*

Aldri  2 – 4 ganger i uken

1 – 3 ganger i måneden  5 ganger eller mer i uken

1 gang i uken

39. **Ost** *[Kryss ett sted]*

Aldri  5 – 6 skiver i uken

1 – 3 skiver i måneden  1 skive om dagen

1 skive i uken  2 eller flere skiver daglig

2 – 4 skiver i uken

40. **Margarin** (mengde som trengs til å smøre en brødskive) *[Kryss ett sted]*

Aldri  1 gang daglig

1 – 3 ganger i måneden  2 – 4 ganger daglig

1 gang i uken  5 ganger eller mer daglig

2 – 6 ganger i uken

41. **Smør** (mengde som trengs til å smøre en brødskive) *[Kryss ett sted]*

Aldri  1 gang daglig

1 – 3 ganger i måneden  2 – 4 ganger daglig

1 gang i uken  5 ganger eller mer daglig

2 – 6 ganger i uken

42. **Peanøttsmør** *[Kryss ett sted]*

Aldri  2 – 4 ganger i uken

1 – 3 ganger i måneden  5 ganger eller mer i uken

En gang i uken

43. **Majones** *[Kryss ett sted]*

Aldri  2 – 4 ganger i uken

1 – 3 ganger i måneden  5 ganger eller mer i uken

En gang i uken

44. **Epler** *[Kryss ett sted]*

Aldri  2 – 4 i uken

1 – 3 i måneden  5 – 6 i uken

Ett i uken  Ett eller flere om dagen

45. **Bananer** *[Kryss ett sted]*

Aldri  2 – 4 per uke

1 – 3 i måneden  5 – 6 per uke

En i uken  En eller flere om dagen

46. **Rosiner** *[Kryss ett sted]*

Aldri  2 – 4 ganger i uken

1 – 3 ganger i måneden  5 – 6 ganger i uken

1 gang i uken  1 ganger eller mer om dagen

47. **Appelsiner** *[Kryss ett sted]*

Aldri  2 – 4 i uken

1 – 3 i måneden  5 eller flere i uken

En i uken  En eller flere om dagen

48. **Appelsinjuice** *[Kryss ett sted]*

Aldri  1 glass daglig

1 – 3 glass i måneden  2 – 3 glass daglig

1 – 4 glass i uken  4 glass eller mer daglig

5 – 6 glass i uken

49. **Eplejuice** *[Sett ett kryss]*

Aldri  1 glass daglig

1 – 3 glass i måneden  2 – 3 glass daglig

1 – 4 glass i uken  4 glass eller mer daglig

5 – 6 glass i uken

50. **Brokkoli** *[Kryss ett sted]*

Aldri  2 – 4 ganger i uken

1 – 3 ganger i måneden  5 ganger eller mer i uken

En gang i uken

51. **Gulrøtter** *[Kryss ett sted]*

Aldri  2 – 4 ganger i uken

1 – 3 ganger i måneden  5 ganger eller mer i uken

En gang i uken

52. **Brekkbønner** *[Kryss ett sted]*

Aldri  2 – 4 ganger i uken

1 – 3 ganger i måneden  5 ganger eller mer i uken

En gang i uken

53. **Erter** *[Kryss ett sted]*

Aldri  2 – 4 ganger i uken

1 – 3 ganger i måneden  5 ganger eller mer i uken

En gang i uken

54. **Mais** *[Kryss ett sted]*

Aldri  2 – 4 ganger i uken

1 – 3 ganger i måneden  5 ganger eller mer i uken

En gang i uken

55. **Spinat** *[Kryss ett sted]*

Aldri  2 – 4 ganger i uken

1 – 3 ganger i måneden  5 ganger eller mer i uken

En gang i uken

56. **Egg** *[Kryss ett sted]*

Aldri  2 – 4 egg i uken

1 – 3 egg i måneden  5 egg eller mer i uken

1 egg i uken

1. **Pølser** *[Kryss ett sted]*

Aldri  2 – 4 ganger i uken

1 – 3 ganger i måneden  5 ganger eller mer i uken

En gang i uken

1. **Kjøttpålegg** (for eks. fårepølse, salami, servelat, bacon) *[Kryss*

*ett sted]*

Aldri  2 – 4 ganger i uken

1 – 3 ganger i måneden  5 ganger eller mer i uken

En gang i uken

1. **Mat av kjøttdeig** (for eksempel kjøttkaker, hamburger, kjøttpudding) *[Kryss ett sted]*

Aldri  2 – 4 ganger i uken

1 – 3 ganger i måneden  5 ganger eller mer i uken

En gang i uken

60. **Kjøtt av storfe, svin, lam** *[Kryss ett sted]*

Aldri  2 – 4 ganger i uken

1 – 3 ganger i måneden  5 ganger eller mer i uken

En gang i uken

61. **Kylling eller kalkun** *[Kryss ett sted]*

Aldri  2 – 4 ganger i uken

1 – 3 ganger i måneden  5 ganger eller mer i uken

En gang i uken

62. **Fisk eller annen sjømat** *[Kryss ett sted]*

Aldri  2 – 4 ganger i uken

1 – 3 ganger i måneden  5 ganger eller mer i uken

En gang i uken

63. **Lever** *[Kryss ett sted]*

Aldri  2 – 4 ganger i uken

1 – 3 ganger i måneden  5 ganger eller mer i uken

En gang i uken

64. **Tomat- eller spagettisaus** *[Kryss ett sted]*

Aldri  2 – 4 ganger i uken

1 – 3 ganger i måneden  5 ganger eller mer i uken

En gang i uken

65. **Pizza** *[Kryss ett sted]*

Aldri  2 – 4 ganger i uken

1 – 3 ganger i måneden  5 ganger eller mer i uken

En gang i uken

66. **Pasta** *[Kryss ett sted]*

Aldri  2 – 4 ganger i uken

1 – 3 ganger i måneden  5 ganger eller mer i uken

En gang i uken

67. **Brød** *[Kryss ett sted]*

Aldri  5 – 7 skiver i uken

1 skive eller mindre i uken  2 – 3 skiver daglig

2 – 4 skiver i uken  4 skiver eller mer daglig

68. **Bakervarer** (for eks. småkaker, kjeks, boller, muffins, wienerbrød og liknende)

*[Kryss ett sted]*

Aldri  2 – 4 ganger i uken

1 – 3 ganger i måneden  5 ganger eller mer i uken

En gang i uken

69. **Ris** *[Kryss ett sted]*

Aldri  2 – 4 ganger i uken

1 – 3 ganger i måneden  5 ganger eller mer i uken

En gang i uken

70. **Frokostblanding** *[Kryss ett sted]*

Aldri  2 – 4 porsjoner per uke

1 – 3 porsjoner i måneden  5 – 7 porsjoner i uken

1 porsjon i uken  2 eller flere porsjoner daglig

71. **Kokte poteter**  *[Kryss ett sted]*

Aldri  2 – 4 ganger i uken

1 – 3 ganger i måneden  5 ganger eller mer i uken

En gang i uken

72. a) Spiste barnet stekte poteter eller pommes frites i førskolealder?

Ja  Nei *(gå til spørsmål 73)*

1. Hvor ofte spiste barnet pommes frites/stekte poteter? *(Kryss ett sted)*

Noen få ganger

1 – 3 ganger i måneden  2 – 4 ganger i uken

En gang i uken  5 eller flere ganger i uken

1. Var potetene vanligvis stekt: *(Kryss ett sted)*

Fra frossen tilstand i pannen eller i stekeovnen

I gatekjøkken eller “fast food” kafe (for eks., McDonald’s eller liknende)

Laget og stekt hjemme *(****Hvis ja,*** *gå til spørsmål 72d)*

1. Dersom du laget pommes frites hjemme, hva slags fett brukte du til stekingen? *(Kryss ett sted)*

Smør  Planteolje

Kyllingfett  Plantemargarin

73. Hvor ofte spiste barnet andre typer stekte poteter (f.eks. potetgull) enn pommes frites? *[Kryss ett sted]*

Aldri  2 – 4 ganger i uken

1 – 3 ganger i måneden  5 ganger eller mer i uken

En gang i uken

74. Hva slags fett brukte du vanligvis til å steke mat (for eks., stekt fisk, kjøtt, kylling)

*[Sett kryss for det som passer]*

Smør  Planteolje

Margarin  Olivenolje

Fast plantefett  Maisolje

Dyrefett  Solsikkeolje

Kyllingfett  Bruker ikke stekefett

75. Pleide barnet å få vitaminpiller i førskolealderen? *(Kryss ett sted)*

Aldri  2 – 4 ganger i uken

1 – 3 ganger i måneden  5 ganger eller mer i uken

En gang i uken

76. Pleide barnet å ta tran i førskolealderen? *(Kryss ett sted)*

Aldri  2 – 4 ganger i uken

1 – 3 ganger i måneden  5 ganger eller mer i uken

En gang i uken

## Del 5: Om barnets aktiviteter

77. Tenk gjennom hva slags aktiviteter barnet ditt drev med i 3-6 årsalderen.

Hvordan vil du beskrive aktivitetsnivået sammenliknet med andre barn på samme alder? *(Kryss ett sted)*

Svært fysisk aktiv (løp og lekte det meste av tiden)

Aktiv

Ikke så aktiv

Lite aktiv (syslet med stillesittende ting for det meste)

78. I 3-6 årsalderen, hvor mange timer daglig brukte barnet å se på TV?

*(Kryss ett sted)*

Så ikke TV  Omtrent 3 timer daglig

Opp til ½ time daglig  4 timer daglig

Omtrent 1 time daglig  5 timer eller mer daglig

Omtrent 2 timer daglig

1. Tenk over aktivitetene til dette barnet etter 6 års alder.

Hvordan vil du beskrive aktivitetsnivået sammenliknet med barn på samme alder?

*(Kryss ett sted)*

Svært fysisk aktiv (løp og lekte det meste av tiden)

Aktiv

Ikke så aktiv

Lite aktiv (leste og drev med andre stillesittende sysler det meste av tiden)

1. I 6-10 årsalder, hvor mange timer daglig brukte barnet å se på TV?

*(Kryss ett sted)*

Så ikke på TV  Omtrent 3 timer daglig

Opp til ½ time daglig  4 timer daglig

Omtrent 1 time daglig  5 timer eller mer daglig

Omtrent 2 timer daglig

1. Har barnet noen gang hatt en av de følgende sykdommene?

*(Kryss Ja eller Nei for hver sykdom)*

a) Diabetes………………………………………………...  Ja  Nei

b) Lungebetennelse……………………………………….  Ja  Nei

c) Urinveisinfeksjon/nyrebekkeninfeksjon………..  Ja  Nei

d) Astma (diagnose satt av lege)………………………  Ja  Nei

e) Allergi i øyne / nese (”høysnue”)……...……………  Ja  Nei

f) Atopisk eksem (”barne-eksem”)…………………….  Ja  Nei

g) Innlagt sykehus, *spesifiser*___________________  Ja  Nei

h) Kreft, *spesifiser*____________________________  Ja  Nei

i) Fjernet mandler………………………………………..  Ja  Nei

j) Annen bakterieinfeksjon, *spesifiser*______________  Ja  Nei

82. a) Bruker barnet noen form for medisiner nå?

Ja  Nei

b) *Hvis ja, vær snill å spesifiser hvilken medisin*

____________________________________

## Del 6: Noen spørsmål om deg

83. Når ble du født?

Dag

Måned

År

**1**

**9**

84. Hva var din fødselsvekt ?

Mindre enn 2.5 kg  Over 4.5 kg

2.5 – 3.9 kg  Vet ikke

4 – 4.5 kg

85. Ble du født i Norge?

Ja  Nei

86. Hva er din ekteskapelige status? *(Kryss ett sted)*

Gift/samboer  Separert  Skilt

Enke  Aldri vært gift eller samboende

87. Hvor høy er du?

Centimeter

88. Dersom du legger på deg, hvor på kroppen vises det best?

*(Kryss av det som passer)*

Rundt brystet og skuldrene

Rundt midjen/magen

Rundt hoftene/lårene

Omtrent likt over det hele

Annet *(Spesifiser)* ________________________________

Legger aldri på meg

89. Hvilken rase tilhører du? *(Kryss av det som passer)*

Europeisk/hvit  Asiatisk

Afrikansk/svart

Latinamerikansk  Annet (*Spesifiser):______________*

90. Hvor gammel var du da du fikk menstruasjon første gang? *(Kryss ett sted)*

Yngre enn 11 år  14 år  18 år eller mer

11 år  15 år  Husker ikke

12 år  16 år

13 år  17 år

91. Hva er som regel lengden på en vanlig menstruasjonssyklus for deg? (antall dager fra første blødningsdag til første blødningsdag i neste syklus) *[Kryss ett sted]*

Mindre enn 21 dager  40 dager eller mer

21 – 25 dager  Svært uregelmessig

26 – 31 dager  Har sluttet å menstruere

32 – 39 dager

1. a) Vi vil gjerne vite mer om alle dine svangerskap som har vart i minst 6 måneder. [G = Gutt; J = Jente]

| **Fødedato**  **(dag/måned/år)** |  | Kjønn |
| --- | --- | --- |
|  | Dødfødt  Levende født | G  J |
|  | Dødfødt  Levende født | G  J |
|  | Dødfødt  Levende født | G  J |
|  | Dødfødt  Levende født | G  J |
|  | Dødfødt  Levende født | G  J |
|  | Dødfødt  Levende født | G  J |

1. Har du noen gang opplevd å ha en spontanabort?

Ja  Nei *(Gå til spørsmål 93)*

Hvor mange ganger?  1  2  3  4  5 eller flere

93. Har du noen gang prøvd å bli gravid i mer enn ett år uten å lykkes?

Ja  Nei

94. Har du eller din partner brukt noen form for prevensjon de siste 12 månedene ?

Ja *(Gå til spørsmål 95)*  Nei

Vil helst ikke svare

*(gå til spørsmål 96)*

95. Kryss av for de prevensjonsmetodene som dere har brukt de siste 12 månedene.

P-pille

P-ring

Hormonsprøyte (”P-sprøyte”)

Hormonimplantat (Implanon®)

Kondomer

Sæddrepende salve, krem eller gele

Pessar

Kobberspiral

Hormonspiral

Sterilisering - kvinnen

Sterilisering (vasektomi) - mannen

“Sikre” perioder

Avbrutt samleie

Andre metoder *(Spesifiser)* ____________________________

96. Er du gravid nå?

Ja

Nei

97. Har du fått fjernet livmoren ?

Ja

Nei

98. a) Har du noen gang hatt forhøyet blodtrykk (målt av lege) uten at du har vært gravid?

Ja  Nei *(Gå til spørsmål 99)*

1. Hvor gammel var du da du fikk diagnosen forhøyet blodtrykk?

________________år

99. a) Har du noen gang fått diagnosen endometriose?

Ja  Nei *(gå til spørsmål 100)*

1. Hvor gammel var du da du fikk diagnosen? ________________år

100. a) Har du noen gang fått diagnosen polycystisk ovariesyndrom (PCO)?

Ja  Nei *(gå til spørsmål 101)*

1. Hvor gammel var du da du fikk diagnosen?________________år

101. a) Har du noen gang hatt brystkreft?

Ja  Nei *(gå til spørsmål 104)*

b) Hvor gammel var du da du fikk diagnosen?________________år

102. Har din biologiske mor hatt brystkreft?

Ja  Nei  Vet ikke

103. a) Hadde din biologiske mor noen gang svangerskapsforgiftning (preeklampsi)?

Ja  Nei *(gå til spørsmål 104)*  Vet ikke

b) Da hun var gravid med deg?

Ja  Nei

c) Da hun var gravid med

en bror av deg

en søster av deg

104. a) Har du noen biologiske søstre? (Ta med avdøde, men ikke ta med halvsøsken)

Ja  Nei *(gå til spørsmål 105)*

1. Hvor mange?

1  3

2  4 eller flere

1. Har noen av dem hatt brystkreft?

Ja  Nei *(gå til spørsmål 105)*  Vet ikke

1. Hvor gammel var hun da hun fikk sykdommen?

Søsters alder  Under 45  50 – 55

45 – 49  56 og eldre

Søsters alder  Under 45  50 – 55

45 – 49  56 og eldre

Søsters alder  Under 45  50 – 55

45 – 49  56 og eldre

e) Vet du om noen av dem hadde preeklampsi (svangerskapsforgifning)?

Ja  Nei *(gå til spørsmål 105)*

f) Hvor mange ganger?  1  2  3  4 eller flere

1. Har du noen gang hatt noen av disse sykdommene?

*(Kryss Ja eller Nei for hver enkelt)*

a) Diabetes…………………………………………. Ja  Nei

b) Polypper i livmoren eller livmorhalsen…………………  Ja  Nei

c) Fibrom i livmoren…………….....………………………… Ja  Nei

d) Hjerteinfarkt ……………………………………………..  Ja  Nei

e) Astma (diagnosert av lege)……………………………... Ja  Nei

f) Godartet cyste / knuter i brystet.......................……….  Ja  Nei

g) Livmorhalskreft………………………………………  Ja  Nei

h) Kreft i livmoren (endometrium)……………………  Ja  Nei

i) Eggstokkreft………………………………………  Ja  Nei

j) Tykktarm- eller endetarmskreft…………………………  Ja  Nei

k) Brystkreft ……………………………………………  Ja  Nei

l) Lungekreft………………………………………………  Ja  Nei

m) Annen kreftsykdom………………………………………  Ja  Nei

106. a) Bruker du noen form for medisin nå?

Ja  Nei

1. Hvis ja, kan du spesifisere

_________________________________________

1. Fylte du ut dette skjemaet ut fra hukommelsen, eller brukte du noen ”hjelpemidler”?: *(Sett kryss for det som passer)*

På egen hånd ut fra hukommelsen

Jeg måtte se etter i babybok, helsestasjonskort eller liknende

Jeg måtte ha hjelp (av partner, slektning, venn)

Takk for hjelpen!
